# Model parameter estimation for Channelrhodopsin-2 light gated ion channels

**DOI:** 10.1186/1471-2202-12-S1-P325

**Published:** 2011-07-18

**Authors:** Shivakeshavan G Ratnadurai, Pramod P Khargonekar, Paul R Carney, Sachin S Talathi

**Affiliations:** 1 Department of Electrical and Computer Engineering, University of Florida, Gainesville, Florida 32611, USA; 2 Department of Biomedical Engineering, University of Florida, Gainesville, Florida 32611, USA; 3 Department of Pedriatics, University of Florida, Gainesville, Florida 32611, USA

## 

Recently, Gunyadin *et al*. [1] designed and validated an engineered mutant of channelrhodopsin-2 (ChR2) labeled E123T to accelerate channel kinetics of ChR2 light gated ion channels. They reported a mono-exponential decay for E123T photocurrent after the light is turned off. This is in contrast to the photocurrent characteristics of the wild type ChR2, which not only exhibits slower channel kinetics, but the decay after the light is turned off is bi-exponential. In earlier work, Nikolic *et al*. [2] had captured the bi-exponential decay of wild type ChR2 in a four state transition rate model.

Our goal in this work is to determine the conditions under which the four state model proposed by Nikolic *et al*. [2] can exhibit fast mono-exponential decay seen for the E123T mutant of ChR2. We start from the four state transition rate model proposed by Nikolic *et al*. [2]. This model consists of two closed dark adapted states C1 and C2 and two open light adapted conducting states O1 and O2. The unknown parameters of the model to be estimated from experimental data are: transition rate parameters: {G_d1_,G_d2_,G_r_, e_12_, e_21_}, parameters ε_1_ and ε_2_ that capture the efficiency of light induced transition of the closed states, and the parameter γ, which captures the relative permeability of the two open states to ion flow. We first identify constraints on the four state model parameters based on the experimental data by Nikolic *et al*. [2] on wild type ChR2 photo-current generated in hippocampal cells for light stimulation with intensities I={0.9, 11} mW/mm^2^. We show that the problem of estimating the unknown parameters defined above from the model derived constraints represents an under-determined system. We convert the parameter estimation problem into a two-step procedure and separate the estimation of light independent and dependent parameters. The parameters are estimated using simulated annealing, a global optimization algorithm. In Table 1, we compare our estimated parameters (MP_ChR2_) for the four state-model with those obtained by Nikolic *et al*. [2]. Our model parameters provide a much better fit to the experimental data on the slow and fast component of the decay current. In particular, Nikolic *et al*. [2] assumed G_r_=0 to derive the constraints on the bi-exponential decay of the ChR2 photocurrent after the light is turned off. We relaxed this assumption and were able to obtain a better fit to the ChR2 decay current kinetics. Specifically, with Nikolic *et al*. [2] parameters, the ratio I_slow_/I_fast_=0.37 for the amplitudes of the slow and fast component of the bi-exponential decay current while I_slow_/I_fast_=0.194, using our parameters. The experimental value reported for this ratio is 0.2. We have further conducted sensitivity analysis on the estimated parameters to understand the degree of variability in the parameters given uncertainty in the experimental data. Using this framework, we then estimate the four state model parameters for E123T mutant of ChR2. We notice that the mono-exponential decay rate of E123T is similar to the fast component of the bi-exponential decay current of wild-type ChR2. This indicates that for the mutant, the amplitude of the slow component of the decay current is zero, which provides one additional constraint on the model parameters. We again use simulated annealing to determine the four state model parameters for the E123T mutant of ChR2. The results of this analysis are presented in row 4 of Table 1. Our analysis of the resulting model reveals that it fits the experimental data from Nikolic *et al*. [2] quite well, with our fit error matching that of Nikolic *et al*. [2] parameters fit error (10E-4).

## Conclusion

We have systematically identified constraints on the four state transition rate model first proposed by Nikolic *et al*. [2] to capture the photocurrent kinetics of both wild type ChR2 with bi-exponential decay rate and E123T mutant with mono-exponential decay rate. Using these constraints and global optimization, we have estimated model parameters that fit experimental data well.
